# *In Vitro* Activity of Cefiderocol, a Siderophore Cephalosporin, against Multidrug-Resistant Gram-Negative Bacteria

**DOI:** 10.1128/AAC.01582-20

**Published:** 2020-11-17

**Authors:** Shazad Mushtaq, Zahra Sadouki, Anna Vickers, David M. Livermore, Neil Woodford

**Affiliations:** a Antimicrobial Resistance and Healthcare Associated Infections Reference Unit, National Infection Service, Public Health England, London, United Kingdom; b Norwich Medical School, University of East Anglia, Norwich, United Kingdom

**Keywords:** *Acinetobacter baumannii*, *Enterobacterales*, *Enterobacteriaceae*, NDM, *Pseudomonas aeruginosa*, antimicrobial activity, antimicrobial resistance, carbapenamase, cefiderocol, multidrug resistance

## Abstract

Cefiderocol is a parenteral siderophore cephalosporin with a catechol-containing 3′ substituent. We evaluated its MICs against Gram-negative bacteria, using iron-depleted Mueller-Hinton broth. The panel comprised 305 isolates of *Enterobacterales*, 111 of Pseudomonas aeruginosa, and 99 of Acinetobacter baumannii, all selected for carbapenem resistance and multidrug resistance to other agents. At 2 and 4 μg/ml, cefiderocol inhibited 78.7 and 92.1%, respectively, of all *Enterobacterales* isolates tested, with rates of 80 to 100% for isolates with all modes of carbapenem resistance except NDM enzymes (41.0% inhibited at 2 μg/ml and 72.1% at 4 μg/ml) or combinations of extended-spectrum β-lactamase (ESBL) and porin loss (61.5% inhibited at 2 μg/ml and 88.5% at 4 μg/ml). Cefiderocol also inhibited 81.1 and 86.5% of all P. aeruginosa isolates at 2 and 4 μg/ml, respectively, with rates of 80 to 100% for isolates with VIM, IMP, GES, or VEB β-lactamases and slightly lower rates for those with NDM (45.5% at 2 μg/ml and 72.7% at 4 μg/ml) and PER (66.7% at 2 μg/ml and 73.3% at 4 μg/ml) enzymes; 63.3% of P. aeruginosa isolates were inhibited at the FDA’s 1-μg/ml breakpoint. Lastly, cefiderocol at 2 and 4 μg/ml inhibited 80.8 and 88.9% of the A. baumannii isolates, respectively, with rates of >85% for isolates with OXA-51-like, -23, -24, or -58 enzymes and 50% at 2 μg/ml and 80% at 4 μg/ml for those with NDM carbapenemases. Dipicolinic acid and avibactam weakly potentiated cefiderocol against *Enterobacterales* isolates with metallo-β-lactamases (MBLs) and serine carbapenemase, respectively, indicating incomplete β-lactamase stability.

## Abstract (precis)

Cefiderocol is a parenteral siderophore cephalosporin with a catechol-containing 3′ substituent. We evaluated its MICs against Gram-negative bacteria, using iron-depleted Mueller-Hinton broth. The panel comprised 305 isolates of *Enterobacterales*, 111 of Pseudomonas aeruginosa, and 99 of Acinetobacter baumannii, all selected for carbapenem resistance and multidrug resistance to other agents. At 2 and 4 μg/ml, cefiderocol inhibited 78.

## INTRODUCTION

Carbapenem-resistant Gram-negative bacteria have proliferated globally and are a growing problem, prioritized by the WHO (1). Their resistance can be caused by acquired carbapenemases or can arise through combinations of porin loss and extended-spectrum or AmpC β-lactamases (2). Carbapenemases are the greater problem insofar as many producers can transfer their β-lactamase genes horizontally. Their enzymes—which variously include KPC, OXA-48-like, and metallo (IMP, NDM, and VIM) β-lactamase (MBLs)—are biochemically diverse, complicating the design of stable β-lactams and inhibitors.

Many carbapenemase producers are resistant to multiple antibiotics besides β-lactams, including fluoroquinolones, aminoglycosides, and antifolates (30). Consequently, clinicians have been forced to redeploy polymyxins, despite doubts about their pharmacokinetics and dose optimization, along with concerns regarding efficacy and renal toxicity (3).

New β-lactamase inhibitor combinations are beginning to provide alternatives, with trials or case series supporting superiority over colistin combinations (4–6). Ceftazidime-avibactam is active against most *Enterobacterales* isolates with KPC and OXA-48-like enzymes, while meropenem-vaborbactam and imipenem-relebactam inhibit those with KPC enzymes only (7, 8). However, none of these combinations are active against *Enterobacterales* isolates with MBLs, nor against the vast majority of carbapenemase-producing isolates of Pseudomonas aeruginosa and Acinetobacter baumannii (9, 10).

Cefiderocol is a novel parenteral siderophore cephalosporin approved in the United State for the treatment for complicated urinary tract infections (cUTI), and in the European Union (EU) for the treatment of infections due to aerobic Gram-negative bacteria in adults with limited treatment options. It has a catechol moiety attached via its 3-position side chain, allowing uptake into Gram-negative bacteria via the ferric iron transporter system (11–13). We evaluated its activity against multidrug-resistant clinical isolates of Gram-negative bacteria from the United Kingdom, prioritizing those with carbapenem resistance (Table 1).

**TABLE 1:** Panel of isolates used in this study

Genus or species	No. of isolates with:																Total
	NDM	VIM	IMP	KPC	OXA-48-like	ESBL + porin loss	AmpC + porin loss	GES	IMI	SME	VEB	PER	OXA-23	OXA-24/40	OXA-51	OXA-58	
*Klebsiella*	20	17	5	20	22	6	7	5									102
E. coli	21	15	5	21	20	11	7										100
*Enterobacter*^*a*^	10	8	4	9	9	8	5	3	5								61
*Serratia*	1			3	2	1	4	1		5							17
*Citrobacter*	3	7	1	3	3												17
*Morganella*	2																2
*Providencia*	3						1										4
*Proteus*	1																1
Hafnia alvei							1										1
P. aeruginosa	11	30	25					20			10	15					111
A. baumannii	20												41	9	19	10	99
Total	92	77	40	56	56	26	25	29	5	5	10	15	41	9	19	10	515

a Includes 8 isolates of Klebsiella aerogenes (formerly Enterobacter aerogenes) and 1 isolate of Pluralibacter gergoviae (formerly Enterobacter gergoviae).

## RESULTS

### Overall MIC distributions.

The isolate panel was deliberately loaded with highly resistant organisms (Table 2), as reflected in the fact that no comparator agent was active against >90% of isolates and only colistin achieved >80% activity across all groups; tigecycline achieved activity versus >90% of *Enterobacterales* isolates based on the FDA 2-μg/ml breakpoint but only 42.0% based on the EUCAST 0.5-μg/ml value, which, strictly, is only applicable to Escherichia coli and Citrobacter koseri.

**TABLE 2:** Overall resistance rates among test panel isolates

Agent(s)	% of isolates at EUCAST or CLSI breakpoint shown in parentheses (μg/ml)					
	*Enterobacterales* (*n* = 305)		P. aeruginosa (*n* = 111)		A. baumannii (*n* = 99)	
	EUCAST R	CLSI not S	EUCAST R	CLSI not S	EUCAST R	CLSI not S
Cefiderocol	21.3 (>2)	7.9 (>4)^*a*^	18.9 (>2)	13.5 (>4)^*a*^	19.2 (>2)^*b*^	11.1 (>4)^*a*^
Meropenem	58.4 (>8)	93.8 (>1)	85.9 (>8)	92.8 (>2)	98 (>8)	99 (>2)
Ceftazidime	87.2 (>4)	97.2 (>4)	99.1 (>8)	99.1 (>8)		95 (>8)
Ceftazidime-avibactam	41.6 (>8)	41.6 (>8)	77.5 (>8)	77.5 (>8)		
Cefepime	79 (>4)	87.2 (>2)	86.5 (>8)	86.5 (>8)		98 (>8)
Ceftolozane-tazobactam	87.2 (>2)	87.2 (>2)	96.4 (>4)	96.4 (>4)		
Aztreonam	71.8 (>4)	72.1 (>4)	53.2 (>16)	66.7 (>8)		
Amikacin	29.5 (>8)	17.7 (>16)	69.4 (>16)	69.4 (>16)	55.6 (>8)	55.6 (>8)
Ciprofloxacin	62 (>0.5)	68.5 (>0.25)	89.2 (>0.5)	89.2 (>0.5)	93.9 (>1)	93.9 (>16)
Tigecycline	42 (>0.5)^*c*^	7.9 (>2)^*d*^		82 (>2)^*d*^		30.3 (>2)^*d*^
Colistin	16.4 (>2)	16.4 (>2)^*e*^	16.2 (>2)	16.2 (>2)^*e*^	11.1 (>2)	11.1 (>2)^*c*^

a Provisional CLSI breakpoint used before licensing: the FDA has since published values of S < 2 μg/ml for *Enterobacterales* (as for EUCAST) and S < 1 μg/ml for P. aeruginosa. A total of 63.3% of the isolates were susceptible at the latter value.

b No EUCAST breakpoint. The proportion susceptible at 2 μg/ml is cited for comparability with other species.

c EUCAST breakpoint of 0.5 μg/ml officially only for E. coli and *C. koseri* adopted for all species.

d FDA breakpoint, as there was no CLSI value.

e No susceptible breakpoint for colistin, so intermediate breakpoint used.

Among comparator β-lactams, ceftazidime-avibactam was the most active combination against *Enterobacterales*, inhibiting 41.6% of isolates at its breakpoint of 8 μg/ml for ceftazidime plus 4 μg/ml for avibactam, essentially comprising almost all those without MBLs. Aztreonam was the most active β-lactam against the P. aeruginosa panel, inhibiting 46.8% of isolates—mostly MBL producers—at its 16-μg/ml breakpoint. Resistance to established β-lactams, including ceftazidime-avibactam, was nearly universal in the A. baumannii panel.

Cefiderocol inhibited 78.7% of *Enterobacterales* isolates at 2 μg/ml and 92.1% at 4 μg/ml; corresponding proportions for the P. aeruginosa collection were 81.1 and 86.5%, respectively, although only 63.3% were inhibited at the FDA’s 1-μg/ml breakpoint. The proportions of the Acinetobacter collection inhibited at 2 and 4 μg/ml were 80.8 and 88.9%, respectively; neither CLSI nor the FDA has breakpoints for this genus.

### *Enterobacterales*.

**FIG 1:**
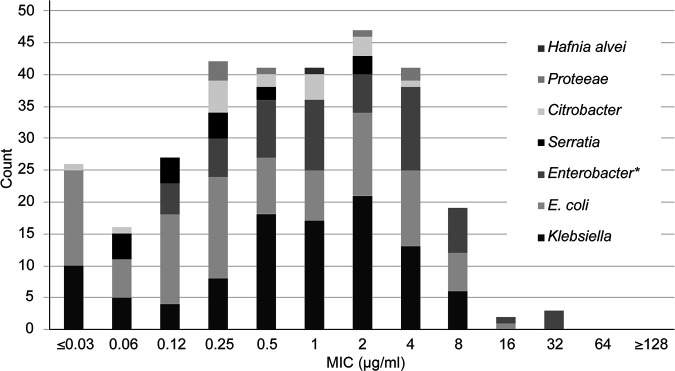
Cefiderocol MIC distribution, by species, for *Enterobacterales* isolates (*n* = 305). An asterisk indicates that the data for *Enterobacter* include 8 isolates of Klebsiella aerogenes (formerly Enterobacter aerogenes) and 1 isolate of Pluralibacter gergoviae (formerly Enterobacter gergoviae).

MICs of cefiderocol were widely scattered within enterobacterial species and had no obvious association with species (Fig. 1). Accordingly, further analysis was done with reference to resistance mechanism rather than species. At 2 μg/ml, cefiderocol inhibited >80% of isolates in all *Enterobacterales* groups except those with NDM carbapenemases (41% inhibited) or combinations of extended-spectrum β-lactamases (ESBLs) and porin loss (61.5% inhibited) (Table 3). At 4 μg/ml, cefiderocol inhibited >95% of isolates in all *Enterobacterales* groups, except for (i) those with NDM MBLs (72.1% inhibited) and (ii) those with combinations of ESBL and impermeability (88.5% inhibited).

**TABLE 3:** MIC distributions of cefiderocol by resistance mechanism and species group

Mechanism	No. of isolates with MIC (μg/ml) of:													% susceptible at MIC (μg/ml) of:	
	≤0.03	0.06	0.125	0.25	0.5	1	2	4	8	16	32	64	≥128	2	4
*Enterobacterales*															
NDM				3	3	6	13	19	13	2	2			41.0	72.1
KPC	9	4	10	7	5	10	6	4	1					91.1	98.2
OXA-48 like	13	5	6	7	10	6	5	3	1					92.9	98.2
VIM	2	1	3	10	9	11	2	7	2					80.9	95.7
ESBL + porin loss			2		6	2	6	7	2		1			61.5	88.5
AmpC + porin loss		2	3	6	4	4	6							100	100
IMP	2	1		6	1		4	1						93.3	100
GES, IMI, or SME		3	3	3	3	2	5							100	100
Total	26	16	27	42	41	41	47	41	19	2	3			78.7	92.1

P. aeruginosa															
VIM	1	2	10	4	6	4	1			1			1	93.3	93.3
IMP		5	4	7	3	1			3	2				80.0	80.0
GES		4	2	5	5	1	1	2						90.0	100
PER		1		2	2	3	2	1	2	2				66.7	73.3
NDM						2	3	3	1	1			1	45.5	72.7
VEB					4	3	2		1					90.0	90.0
Total	1	12	16	18	20	14	9	6	7	6			2	81.1	86.5

A. baumannii															
OXA-23		2	11	11	5	3	3	1		1		1	3	85.4	87.8
NDM						3	7	6	1	1			2	50.0	80.0
OXA-51		5	7	5	1					1				94.7	94.7
OXA-58		2	3	4									1	90.0	90.0
OXA-24/40				1	3	2	2	1						88.9	100
Total		9	21	21	9	8	12	8	1	3		1	6	80.8	88.9

Irrespective of species, the MIC distribution for *Enterobacterales* isolates with NDM enzymes was extended and elevated compared with those for isolates with other MBLs. This behavior was independent of aztreonam resistance, indicating that higher cefiderocol values, where seen, were not contingent upon coresident ESBL or AmpC enzymes (Table 4).

**TABLE 4:** Distributions of cefiderocol versus aztreonam MICs for *Enterobacterales* isolates with NDM carbapenemases^*a*^

Aztreonam MIC (μg/ml)	No. of isolates with cefiderocol MIC (μg/ml) of:								Total
	0.25	0.5	1	2	4	8	16	32	
≤0.5	1	2	2	7	4	2		1	19
1					2	1			3
2	1				2	1	1		5
4				1	2				3
8					1				1
16									0
32		1		1	1	2		1	6
≥64	1		4	4	7	7	1		24
Total	3	3	6	13	19	13	2	2	61

a *n* = 61 isolates.

### P. aeruginosa.

At 2 μg/ml, cefiderocol inhibited 81.1% of P. aeruginosa isolates tested, with rates between 90.0 and 93.3% for all groups, except for those with IMP (80.0%), PER (66.7%), and NDM (45.5%) β-lactamases (Table 3). At 4 μg/ml, cefiderocol inhibited 86.5% of P. aeruginosa isolates tested, with rates between 90.0 and 100% for all resistance mechanism groups, except for those with IMP (80.0%), PER (73.3%), or NDM enzymes (72.7%). Cefiderocol MICs of ≥128 μg/ml were recorded for two isolates: one with an NDM carbapenemase and one with a VIM carbapenemase.

As with *Enterobacterales*, cefiderocol MICs for P. aeruginosa isolates with NDM carbapenemases were elevated compared with those for isolates with other mechanisms, although it should be cautioned that (i) the behavior seemed less marked and (ii) only 11 P. aeruginosa isolates with NDM carbapenemases were tested—far fewer than the 61 *Enterobacterales* isolates.

### A. baumannii.

At 2 μg/ml, cefiderocol inhibited 80.8% of the test panel of A. baumannii isolates, with rates of ≥85% for all groups, except for those with NDM carbapenemases (50.0%); at 4 μg/ml, it inhibited 88.9% (Table 2), with rates of ≥87% for all groups, except for those with NDM carbapenemases (80.0%) (Table 3). Nevertheless, MICs of ≥64 μg/ml were recorded for 7 of the 99 isolates, comprising two with NDM carbapenemases, four with OXA-23, and one with OXA-58.

MIC distributions for A. baumannii with NDM or OXA-23 enzymes were elevated compared with those for isolates with other mechanisms. A caveat to note: although Table 3 indicates that 10% of A. baumannii isolates with OXA-58 carbapenemases were resistant to cefiderocol at 128 μg/ml, this represents only a single isolate, meaning that significance is limited.

### Cefiderocol combined with β-lactamase inhibitors.

Because cefiderocol MICs for isolates with NDM carbapenemases were generally higher than those for isolates with other carbapenemases, we sought to explore whether these isolates could inactivate the compound. To do this, we redetermined MICs of cefiderocol together with dipicolinic acid (100 μg/ml), as an inhibitor of MBLs, and avibactam (4 μg/ml), as an inhibitor of any coproduced serine β-lactamases, and with a combination of both these inhibitors. A panel of 40 *Enterobacterales* isolates with NDM enzymes was used together with small control groups representing other carbapenemases or mechanisms (Table 5).

**TABLE 5:** MICs of cefiderocol for 64 *Enterobacterales* isolates determined with and without β-lactamase inhibitors

lnhibitor and agent(s)^*a*^	MIC (μg/ml)										
	≤0.03	0.06	0.125	0.25	0.5	1	2	4	8	16	32
NDM (*n* = 40)											
Cefiderocol						5	4	13	12	4	2
+ avibactam					2	3	6	12	10	5	2
+ dipicolinic acid	1		3	2	5	11	7	8	2	1	
+ avibactam and dipicolinic acid	1	3	2	8	6	15	5				

VIM (*n* = 6)											
Cefiderocol		1						2	3		
+ avibactam		1					1	3	1		
+ dipicolinic acid		2					2	2			
+ avibactam and dipicolinic acid	1			1	1	2	1				

KPC (*n* = 5)											
Cefiderocol							2	2		1	
+ avibactam					1	4					
+ dipicolinic acid							3	1	1		
+ avibactam and dipicolinic acid					2	3					

OXA-48 like (*n* = 4)											
Cefiderocol				1		1	2				
+ avibactam		1		1	1	1					
+ dipicolinic acid					1	1	1	1			
+ avibactam and dipicolinic acid			1	2		1					

ESBL + porin loss or AmpC + porin loss (*n* = 8)											
Cefiderocol						1	1	4	2		
+ avibactam		1	3		1	2	1				
+ dipicolinic acid					1	2	1	2	1		1
+ avibactam and dipicolinic acid		1		4	1	1	1				

a *n* indicates the number of isolates.

Avibactam alone had predictably little effect on the MICs of cefiderocol for the isolates with NDM enzymes: only 1/40 NDM-positive *Enterobacterales* isolates showed a >2-fold reduction in cefiderocol MIC. Dipicolinic acid, in contrast, achieved 4- to 32-fold reductions in MIC for 25/40 *bla*_NDM_-positive isolates, with combination MICs ranging from ≤0.03 to 16 μg/ml and a mode of 1 μg/ml, compared with 4 μg/ml for cefiderocol alone. There was a greater effect when both inhibitors were tested together in combination with cefiderocol, with 4- to 64-fold MIC reductions for 36/40 *bla*_NDM_-positive isolates. Using this triple combination, all 40 NDM-positive isolates were inhibited by cefiderocol at ≤2 μg/ml compared with 22.5% for cefiderocol alone.

The control groups were small, reducing the strength of conclusions for individual enzyme types: nonetheless, MICs of cefiderocol for isolates with VIM enzymes generally showed 2- to 4-fold reductions when dipicolinic acid was added alone or together with avibactam, whereas no reductions were seen with only avibactam added. Conversely, the MICs of cefiderocol for isolates with KPC or OXA-like carbapenemases, or with combinations of ESBL and porin loss, mostly were reduced by the addition of avibactam alone or combined with dipicolinic acid, whereas dipicolinic acid alone had minimal effect.

## DISCUSSION

Cefiderocol is the first catechol β-lactam to be licensed. It combines efficient entry into Gram-negative bacteria with considerable β-lactamase stability. These factors support *in vitro* activity against otherwise extremely resistant *Enterobacterales* isolates and nonfermenters, at least under iron-deficient conditions. Thus, as found here and by others (12–14), cefiderocol inhibited the majority of carbapenem-resistant Gram-negative bacteria, tested irrespective of species, at 2 or 4 μg/m. This activity encompassed many MBL-producing isolates of *Enterobacterales*, P. aeruginosa, and A. baumannii, as well as isolates of A. baumannii with OXA carbapenemases, whereas these groups typically are unequivocally resistant to new β-lactamase inhibitor combinations, including ceftazidime-avibactam (as confirmed here), meropenem-vaborbactam, and imipenem-relebactam (15–17).

These aspects are positive, but three interlinked areas of uncertainty remain: the “correct” breakpoints, the activity against bacteria with NDM carbapenemases, and the clinical efficacy against carbapenemase producers. The breakpoint issues have been recently and well summarized by Simner and Patel (18). Succinctly, a provisional CLSI value of susceptible (S) ≤ 4 μg/ml and resistant (R) > 4 μg/ml was adopted in development and has been retained as a reference point here; however, EUCAST has subsequently advised breakpoints of S < 2 μg/ml and R > 2 μg/ml for all species, and the FDA has adopted values of S < 2 μg/ml and R > 4 μg/ml for *Enterobacterales* and S ≤ 1 μg/ml and R > 2 μg/ml for P. aeruginosa, with the last of these values predicated on a cUTI trial (19) where there was only a single P. aeruginosa isolate with a MIC of >1 μg/ml. It is not now clear whether CLSI will retain their provisional values or adopt the lower FDA values; a decision is anticipated in 2021 (18).

These breakpoint issues become particularly pertinent for bacteria with NDM carbapenemases, as the MICs of cefiderocol for these mostly were higher than those for isolates of the same species with other carbapenemases. In particular, cefiderocol MICs were >4 μg/ml for 17/61 (27.9%) of *Enterobacterales* isolates with NDM carbapenemases compared with 2/62 (3.2%) of those with VIM and IMP MBLs (*P* < 0.001, chi-square test). This behavior was unrelated to aztreonam resistance or susceptibility, implying that the higher MICs reflected the NDM enzymes themselves and not coproduced ESBLs or AmpC enzymes. To explore this aspect further and to determine if NDM enzyme protected bacteria against cefiderocol, we undertook combination tests with β-lactamase inhibitors. The results supported the view that cefiderocol is not completely β-lactamase stable, as it was frequently, though weakly, potentiated by dipicolinic acid against the isolates with NDM (particularly) and VIM MBLs and by avibactam for isolates with KPC, OXA-48, ESBL, and AmpC enzymes. Additional synergy for *Enterobacterales* isolates with NDM enzymes when both dipicolinic acid and avibactam were added is surprising. It may be that avibactam inhibits coresident class A or D β-lactamases, but were this a significant factor, one would expect the MICs of unprotected cefiderocol to be higher for aztreonam-resistant, NDM-positive *Enterobacterales* isolates than for their aztreonam-susceptible counterparts lacking ESBLs or AmpC enzymes, and this was not the case (Table 4).

The significance of this slight β-lactamase lability is difficult to judge: imipenem remains clinically useful against P. aeruginosa, unless OprD is lost, despite lability to the organism’s chromosomal AmpC enzyme (20); on the other hand, breakpoints for oxyimino cephalosporins against *Enterobacterales* have had to be lowered substantially because even modestly raised values have been associated with clinical failures when ESBLs are present (21).

Relating these MIC observations to clinical data is presently difficult. Recent phase III trials have shown cefiderocol to be effective and noninferior to imipenem-cilastatin for the treatment of cUTIs and to meropenem in nosocomial pneumonia caused by Gram-negative bacteria (19, 22). However, these trials mostly recruited patients with broadly susceptible pathogens, and a further study, CREDIBLE-CR (23, 24), gave more equivocal results. This compared cefiderocol with “best available therapy”—comprising colistin or its combinations in 66% of cases—in multiple infection types involving carbapenem-resistant Gram-negative bacteria. Overall, cefiderocol achieved comparable clinical and microbiologic outcomes to its comparators, but disturbingly, there was a significant excess of deaths in the cefiderocol arm, many involving infections with Acinetobacter spp. Formal publication and analysis of these data are awaited, and the issue of outcomes relative to enzyme type as well as species and MIC will be of vital importance. More positively, several case reports have appeared describing the successful use of cefiderocol as compassionate therapy in infections involving difficult extremely resistant pathogens (25–27).

Ultimately, accumulating clinical experience will determine cefiderocol’s utility against carbapenemase-producing Gram-negative bacteria. Given the drug’s unusual mode of uptake, this utility may depend on the degree of iron starvation that applies at a particular infection site. What can fairly be said at this stage is that its MICs for many isolates that are resistant to other new agents are sufficiently low to permit a degree of guarded optimism.

## MATERIALS AND METHODS

### Bacteria.

The test isolates (Table 1) comprised (i) 305 isolates of *Enterobacterales*, selected to represent diverse carbapenemase producers and isolates with carbapenem resistance via combinations of porin loss with AmpC or ESBL activity; (ii) 111 isolates of P. aeruginosa, selected to represent producers of MBLs and GES carbapenemases, along with isolates that produced VEB or PER ESBLs and were carbapenem resistant via OprD loss; and (iii) 99 isolates of A. baumannii with NDM MBLs or various OXA carbapenemases.

In selecting isolates from Public Health England’s Antimicrobial Resistance and Healthcare Associated Infections (PHE-AMRHAI) Reference Unit collections for inclusion, we favored organisms with phenotypes for other β-lactams (cefiderocol had not previously been tested), typical of their mechanisms but including susceptible and resistant representatives for antibiotics that divide isolates within groups. Thus, for example, groups with metallo-β-lactamases were selected to include both aztreonam-resistant organisms (inferred also to have ESBLs or AmpC enzymes) and those that were aztreonam susceptible (inferred to lack ESBLs or AmpC enzymes). We ensured that the isolates were from diverse hospitals and excluded multiple isolates from single patients. Almost all of the organisms were submitted by hospital laboratories in the United Kingdom between 2008 and 2018 for investigation of unusual resistance phenotypes and/or susceptibility testing for therapeutic guidance. Exceptions were 3 isolates referred to the PHE-AMRHAI Reference Unit from hospitals in the Republic of Ireland and 11 isolates of Pseudomonas aeruginosa with PER ESBLs, which were collected in Turkey in the early 1990s (28).

Carbapenemases and VEB and PER enzymes were identified by PCR of their encoding genes or by whole-genome sequencing (WGS). Carbapenem resistance due to porin loss combined with ESBL or AmpC activity was inferred from previous susceptibility results and the absence of carbapenemase, as confirmed by PCR or WGS. Species identification was by matrix-assisted laser desorption ionization–time of flight (MALDI-TOF) mass spectroscopy.

### MIC testing.

MICs were determined using preprepared broth microdilution plates (IHMA, Inc., Schaumburg, IL) with antibiotic dilutions in cation-adjusted Mueller-Hinton broth (CAMHB) (29). Iron-depleted CAMHB (ID-CAMHB) was used for cefiderocol only and was prepared by IHMA, by treating CAMHB with a cation-binding resin (Chelex; Bio-Rad), followed by removal of resin by filtration and addition of Mg^2+^, Ca^2+^, and Zn^2+^ ions at concentrations of 20 to 25, 10 to 12.5, and 0.5 to 1.0 μg/ml, respectively. The comparator antibiotics were meropenem, ceftazidime, ceftazidime-avibactam (4 μg/ml), cefepime, ceftolozane-tazobactam (4 μg/ml), aztreonam, colistin, amikacin, ciprofloxacin, and tigecycline, all sourced by IHMA.

A subpanel of 64 *Enterobacterales* isolates was similarly tested on a second broth microdilution plate that included cefiderocol alone, cefiderocol-avibactam (4 μg/ml), cefiderocol-dipicolinic acid (100 μg/ml), and cefiderocol plus both avibactam (4 μg/ml) and dipicolinic acid (100 μg/ml). ID-CAMHB was used.

We reviewed results against published breakpoints: EUCAST has values of S ≤ 2 μg/ml and R > 2 μg/ml for *Enterobacterales* and P. aeruginosa; the FDA has values of S ≤ 2 μg/ml and R > 4 μg/ml for *Enterobacterales* and S ≤ 1 μg/ml and R > 2 μg/ml for P. aeruginosa; CSLI still has values under review, but previously, when cefiderocol was in trial, had investigational values of S ≤ 4 μg/ml and R > 8 μg/ml. MICs of comparator antibiotics were interpreted using EUCAST guidelines where available, the exceptions being ceftazidime and cefepime for Acinetobacter spp., for which only CLSI breakpoints are available.
